# Transforming early cancer detection in primary care: harnessing the power of machine learning

**DOI:** 10.18632/oncoscience.578

**Published:** 2023-06-09

**Authors:** Elinor Nemlander, Marcela Ewing, Axel C. Carlsson, Andreas Rosenblad

**Keywords:** Primary care, Early cancer detection, Machine learning, Risk assessment

## INTRODUCTION

Cancer remains a significant global health burden, and early detection plays a crucial role in improving patient outcomes. Primary care settings serve as frontline gatekeepers, providing an opportunity for early detection through symptom assessment and targeted screening. However, detecting early-stage cancer and identifying individuals at high risk can be challenging due to the complexity and subtlety of symptoms [1]. The challenging nature of early detection is revealed by diagnostic errors in primary care, with cancer being one of the most frequently missed or delayed diagnoses [2].

In recent years, the emergence of machine learning (ML) techniques has shown promise in revolutionizing early detection efforts [3]. This editorial explores the potential of ML in enhancing early cancer detection in primary care.

### Harnessing the power of machine learning for early detection

ML algorithms use statistical models to analyse data and have the capability to process large amounts of patient data, identify pre-diagnostic patterns, and generate predictive models. In the context of early cancer detection, ML can leverage diverse data sources, including electronic health records, laboratory analyses, lifestyle factors, and patient-reported symptoms, to develop robust risk assessment and pre-diagnostic tools. These tools require structured medical data but can help clinicians detect patterns, signs and combinations of symptoms that are not discernible for the human brain.

By analysing multidimensional datasets, ML algorithms can uncover hidden associations, better recognize pre-diagnostic patterns, reduce bias in pre-diagnostic assessments, identify high-risk individuals, and thereby minimize the risk of missing cancer diagnoses. This approach could optimize the allocation of healthcare resources, ensuring that high-risk individuals receive timely and appropriate interventions.

Using ML algorithms in risk assessment introduces some critical aspects to consider: the risk of overfitting, maintaining algorithm transparency, mitigating potential biases, and ensuring model generalizability. These considerations are essential to ensure trustworthiness and enhance the applicability and effectiveness of ML models. To achieve this, it is vital to develop and train these models using representative datasets that accurately reflect the target population and the specific context where the models can be deployed [4].

### The use of diagnostic coding

General practitioners often have access to extensive patient data; however, the data are not consistently documented in a structured manner that facilitates ML analyses. Inconsistent data represent a trade-off between the desire for structured data and the time spent with patients, as implementing a structured collection of data may require additional administrative efforts that could distract from patient care, given the high demands and workloads in already pressured primary care settings.

Diagnostic coding, such as ICD codes, are collected in a structured way. Although diagnostic coding is commonly used, there are limitations in the objectivity and completeness of these codes. Most healthcare systems have reimbursement systems that partially rely on patients’ illness severity, which may influence the coding practices. Studies have also indicated that primary care physicians may not code all symptoms reported by patients [5]. This raises questions regarding the selective nature of symptom coding and whether the physician’s assessment of a symptom’s relevance to cancer or other diseases influences the decision to code it. In such cases, it becomes important to consider whether a risk assessment tool based on symptom coding primarily reflects the clinician’s ability to discern pertinent symptoms from those that are less relevant.

Another aspect is the information carried by the accumulated patient’s diagnoses. The impact of chronic diagnoses on cancer has been shown to be important in previous studies for both cancer risk [6], and cancer detection [7, 8], but those diagnoses are not coded annually for all patients in primary health care [9].

The differences in coding traditions within and between countries are not well known [10]. The methods for weighing different diagnoses in predictive models and considering different coding practices in risk assessments are still uncertain and should be further explored.

The process of diagnostic coding typically takes place after the patient’s visit to the clinic, which raises concerns regarding the effectiveness of a tool that alerts primary care clinicians to a patient’s elevated risk of cancer after the patient has already left the clinic. It is, however, important to note that these algorithms are not intended as standalone diagnostic tools; rather, they are designed to assist in identifying patients who may be at risk and require further testing. Therefore, their optimal utilization involves a combination of clinical judgment and additional diagnostic tests to reach a definitive diagnosis.

To improve the effectiveness of risk assessment models, it is important to explore the incorporation of more objective measurements, such as structured laboratory results. ML techniques have already shown promise in generating personalized cancer risk scores using routine laboratory tests administered when general practitioners refer patients with non-specific symptoms [11].

### Challenges and considerations

While ML holds big potential, several challenges need to be addressed. Data privacy, algorithm transparency, and the potential for biases in algorithm development are critical concerns. It is essential to validate algorithms across diverse populations and collaborate between researchers, clinicians, and policymakers to establish standardized protocols and ensure equitable access to these innovative tools. Additionally, the choice of the appropriate method, whether ML or traditional methods, should be determined by the specific task at hand, as no single approach is universally superior.

## CONCLUSION

ML has the potential to transform early cancer detection in primary care by leveraging extensive patient data and improving risk stratification and pre-diagnostic accuracy, hopefully saving lives. However, responsible, and equitable implementation of ML models requires careful attention to ethical considerations, collaboration, and validation across diverse populations.
